# Patient-reported outcomes measurement information system instruments in knee arthroplasty patients: a systematic review of the literature

**DOI:** 10.1186/s43019-023-00201-6

**Published:** 2023-12-01

**Authors:** Natalia Czerwonka, Puneet Gupta, Sohil S. Desai, Thomas R. Hickernell, Alexander L. Neuwirth, David P. Trofa

**Affiliations:** 1 Department of Orthopedic Surgery, Columbia University Irving Medical Center, New York-Presbyterian Hospital, 622 W 168th St., PH11-Center Wing, New York, NY 10032 USA; 2 George Washington University School of Medicine, 2300 I St NW, Washington, DC 20052 USA

**Keywords:** PROMIS, Total knee arthroplasty, Patient-reported outcomes, MCID

## Abstract

**Background:**

The purpose of this study is to provide a systematic review of the literature pertaining to Patient-Reported Outcome Measurement Information System (PROMIS) validation and utilization as an outcomes metric in total knee arthroplasty (TKA) patients. This is the first systematic review on PROMIS use in total knee arthroplasty patients.

**Methods:**

A systematic search of the Pubmed/MEDLINE and Embase databases was performed according to the Preferred Reporting Items for Systematic Reviews and Meta-Analyses (PRISMA) guidelines. Study characteristics, patient demographics, psychometric properties (Pearson and Spearman correlation) with legacy patient-reported outcome measurement (PROM) instruments, floor and ceiling effects, responsiveness, and minimum clinically important difference (MCID) and PROMIS outcomes were recorded and analyzed.

**Results:**

Fifteen studies investigating PROMIS in 11,140 patients were included. The weighted-average Pearson correlation coefficient comparing PROMIS domains with legacy patient-reported outcome measurements in total knee arthroplasty patients was 0.62 [standard error (SE) = 0.06] and the weighted-average Spearman correlation comparing PROMIS domains with legacy patient-reported outcome measurements in total knee arthroplasty patients was 0.59 (SE = 0.06), demonstrating moderate-to-strong correlation and validity. There were no differences in weighted average floor [0.03% (SE = 3.1) versus 0% (SE = 0.1) versus 0.01% (SE = 1.1); *p* = 0.25] or ceiling effects [0.01% (SE = 0.7) versus 0.02% (SE = 1.4) versus 0.04% (SE = 3.5); *p* = 0.36] between PROMIS and legacy instruments. The weighted average for percentage of patients achieving MCID was 59.1% for global physical health (GPH), 26.0% for global mental health (GMH), 52.7% for physical function (PF), 67.2% for pain interference (PI), and 37.2% for depression.

**Conclusion:**

Notably, PROMIS global physical health, physical function, and pain interference were found to be significantly responsive, with PROMIS pain interference most effectively capturing clinical improvement as evidenced by the achievement of MCID.

## Main text

### Introduction

Patient-reported outcome measures (PROMs) are validated and standardized questionnaires completed by patients that evaluate a patient’s insight into their quality of health, return to baseline function, and mental well-being [1]. Within the field of knee arthroplasty, commonly used PROMs include the Western Ontario and McMaster Universities Osteoarthritis Index (WOMAC), the Knee Injury and Osteoarthritis Outcomes Score (KOOS), the Oxford Knee Score (OKS), and the Lower Extremity Activity Scale (LEAS). These validated PROMs are collectively referred to as legacy instruments. However, completion of PROMs can be a challenging technological and administrative enterprise [2–4]. Additionally, lack of standardization among PROMs leads to multiple PROM questionnaires being administered per patient; this can increase the potential for “survey question fatigue” and resultant data incompleteness [5–7]. Further, Sabah et al. demonstrated that though there are eight joint-specific PROMs available to evaluate knee replacement outcome scores, only three of these (KOOS, LEAS, and WOMAC) have sufficient evidence of validity [8]. Overall, financial barriers, administrative constraints, and lack of standardization have been cited as obstacles to effective PROM collection and utilization. [5–7]

The Patient-Reported Outcome Measurement Information System (PROMIS) is an envisioned gold-standard outcome measurement tool intended to provide psychometrically standardized and validated patient-reported outcomes [9]. PROMIS measures a patient’s physical and mental health across multiple domains such as pain intensity (PROMIS Pain Intensity), pain interference (PROMIS PI), and physical function (PROMIS PF) [10]. Pain interference assesses the effects of pain on the important aspects of one’s life, such as the extent to which pain prevents engagement with emotional, social, physical, and cognitive activities. Likewise, PROMIS global domains such as global mental health (GMH) and global physical health (GPH) assess the overall state of one’s mental and physical health, respectively. It is administered in two forms: a computer adaptive test (CAT) and short form. Based on the item response theory (IRT), the CAT test is a sequence of consecutive questions tailored for delivery based on real-time patient responses, thus allowing for streamlined questioning based on the subject’s previous responses. Studies have shown CAT to be more time-efficient than traditional legacy instruments, requiring fewer questions to reach the identical level of responsiveness [11, 12]. Other advantages of PROMIS include its validity, responsiveness, coverage, and decreased floor and ceiling effects compared with legacy instruments across various orthopedic patient populations. [13–15]

To support widespread implementation of PROMIS within the practice of specific orthopedic subspecialties and in the perioperative periods of specific procedures, it is important to evaluate the validity and current utilization of these instruments. The purpose of this study is to provide a systematic review of the literature pertaining to PROMIS validation and utilization as an outcome metric in total knee arthroplasty (TKA) patients.

## Methods

This systematic review was performed according to the Preferred Reporting Items for Systematic Reviews and Meta-Analyses (PRISMA) guidelines [16]. The PROSPERO database was searched by two of the authors (NC, PG) for existing systematic reviews on the present topic. Pubmed/MEDLINE and Embase databases were searched using the search terms (“patient-reported outcomes measurement information system” OR “PROMIS”) AND (“knee”) AND (“arthroplasty”) to identify all relevant literature. Inclusion and exclusion criteria for the resultant abstracts were applied once duplicates were excluded. Two independent reviewers evaluated the identified abstracts; if no consensus was reached regarding the choice to include or exclude a particular study, the senior author was consulted, and a consensus was reached. For each included article, the references were manually scanned for additional papers not identified in the original database search. As this was a systematic review of published literature, institutional review board approval was not required.

**Fig. 1:**
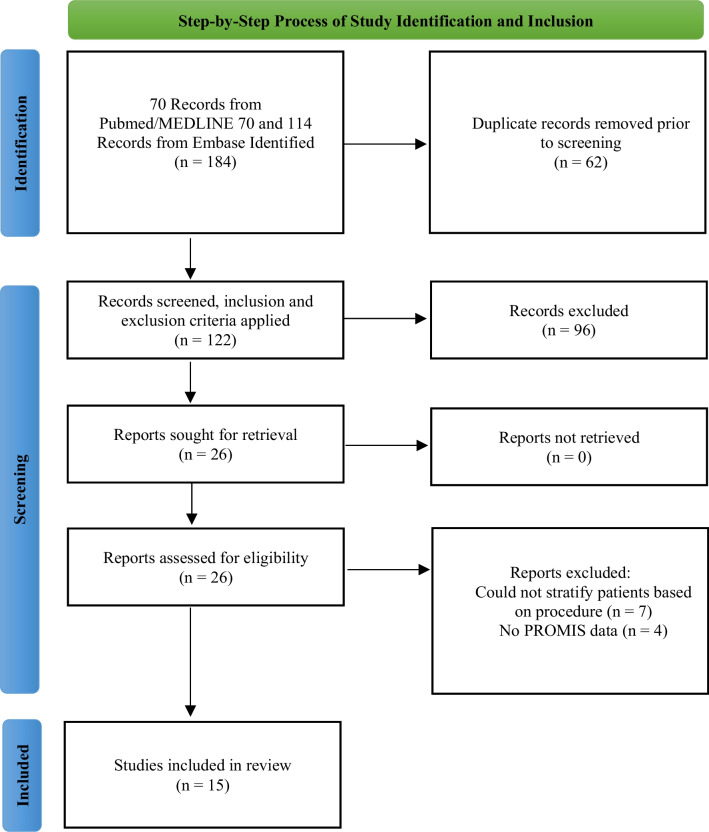
Step-by-step process of study identification and inclusion

Published articles reporting on PROMIS in TKA patients were identified (Fig. 1). The inclusion criteria were as follows: any study with level of evidence I–IV that reported the psychometric properties [Pearson and Spearman correlation with legacy instruments, floor and ceiling effects, responsiveness, and minimum clinically important difference (MCID)] and/or utilization of PROMIS in total knee arthroplasty. Study characteristics, patient demographics, psychometric properties, and PROMIS outcomes were recorded and analyzed. Exclusion criteria were as follows: level of evidence V, conference abstracts, reviews, articles written in non-English languages without translation available, and studies that did not distinguish between TKA or total hip arthroplasty (THA), revision TKA, unicompartmental knee arthroplasty, or general knee surgery. Studies were further excluded if PROMIS data were not able to be directly extracted.

The following information was collected: level of evidence, study design, number of patients, mean age of patients, and gender distribution. Pearson’s *r* and Spearman’s rho were collected to assess the correlation between PROMIS and legacy measures, which included: KOOS Joint Replacement (KOOS-JR), KOOS Functions in Activities of Daily Living (KOOS-ADL), KOOS Sports, KOOS Physical Function Short Form (KOOS-PS), Brief Resilience Scale (BRS), OKS, and modified single assessment evaluation (M-SANE). In this systematic review, very weak correlation is defined as *r* = 0–0.19, weak as *r* = 0.20–0.39, moderate as *r* = 0.40–0.59, strong as *r* = 0.60–0.79, and very strong as *r* = 0.80–1.00. Floor and ceiling effects were recorded to assess the coverage (the ability of an instrument to detect the full range of scores of a given measure in a patient population). The generally accepted benchmark for which floor and ceiling effects are considered significant ranges from 5% to 15% of participants scoring the minimum or maximum scores [17, 18]. Responsiveness was evaluated through recorded preoperative and postoperative PROMIS and legacy PROM scores. The established MCID values per PROMIS domain and legacy instrument, as well as the percentage of patients who achieved MCID, was also collected. MCID is the smallest change in scores on a given instrument that represents a clinically important difference to the patient [19]. In general, a smaller MCID is desirable, as that indicates that the change in score is real and not attributed to measurement error.

Studies that did not include analysis of the psychometric properties of PROMIS but reported on PROMIS as an outcome and predictive metric in TKA were also included. Additional data such as patient population, intervention, follow-up time, primary assessment, and main PROMIS findings were collected from each article.

### Methodological quality and risk of bias assessment

Two authors (NC, PG) applied the Methodological Index for Non-Randomized Studies (MINORS) criteria to each study included in the systematic review to assess the methodological quality [20]. Cohen’s kappa values were computed to analyze inter-reviewer reliability for each item of the MINORS criteria and assess the degree of concurrence between the two blinded reviewers. The highest attainable score is 24, which denotes that the study under scrutiny is of good methodological quality.

Multiple steps were taken to avoid publication bias, such as including studies that reported both positive and negative results, applying the MINORS criteria to each included study.

### Statistical analysis

Weighted averages for the following metrics were calculated: the correlation of PROMIS with legacy instruments (via Pearson and Spearmen correlation coefficients, in which absolute values of coefficients were used); floor and ceiling effects; preoperative, < 3 months postoperative, and ≥ 6 months postoperative PROMIS scores (to assess responsiveness); and MCID scores. The study sample size was used to assign weights. One-way ANOVA and student *t*-tests were used to determine statistically significant differences. All statistical analyses were carried out in Microsoft Excel Version 16.72, with a cutoff for statistical significance at *p* < 0.05. Meta-analysis of correlations (https://data-play3.shinyapps.io/Meta_corr/) software was used for meta-analysis, with application of a fixed-effects model to determine whether a statistically significant difference existed between the correlations of PROMIS to legacy instruments between studies.

## Results

The preliminary database search yielded 184 studies. Once duplicates were removed, the remaining 122 studies were screened and 15 studies investigating PROMIS in 11,140 patients were ultimately included in this systematic review (Fig. 1; Table 1) [21–35].

**Table 1:** Inclusion and exclusion criteria of total knee arthroplasty studies encompassed in this review

Study	Date	LOE	Patients (*n* =)	Procedure	Mean age (year)	Sex (% M)	Inclusion	Exclusion
*Validation*								
Khalil et al. [19]	Jan 2020	III	875	TKA	67.5	NR	Completion of at least one preoperative and one postoperative set of surveys	Bilateral staged TKAs, non-English speakers, or revision TKA
Padilla et al. [20]	Oct 2018	III	124	TKA	65.2	34.7	Age > 18 years, completed PROM surveys and reported knee pain with subsequent TKA	Bilateral TJA, partial joint arthroplasty (UKA), or revision arthroplasty
Heng et al. [21]	June 2021	III	1003	TKA	67	40	Age > 18 yerars, TKA	No PROMs collected, only one PROM completed
Austin et al. [22]	July 2019	III	217	TKA	NR	43	All patients undergoing TKA	No PROMs collected, only one PROM completed
Shim et al. [23]	July 2019	III	721	TKA	69		OA, all patients undergoing TKA	NR
Shaw et al. [24]	Feb 2021	II	1160 (260 rTKAs, 900 standard TKA)	TKA (standard manual or robotic)	rTKA: 67.2 TKA: 67.1	rTKA: 32.3 TKA: 30.4	Robotic or standard manual TKA, completed at least one preoperative and one postoperative PROM survey	Partial joint arthroplasty, arthroplasty secondary to trauma, revision arthroplasty, and multiple arthroplasty procedures within selected study period
Darrith et al. [25]	Dec 2021	III	872	TKA	67.5	NR	Primary unilateral TKA	Bilateral TKA, revision TKA, no reported outcome measurements
Nwanko et al. [26]	July 2005	II	404	TKA	62	47	Primary unilateral TKA, ages 35–85 years, English speaking, able to provide informed consent	Medically unstable at presentation, TKA due to fracture, malignancy, bilateral TKA, infection, cognitive or neurologic disorder
Stiegel et al. [27]	May 2019	II	50	TKA	NR	36	Primary TKA or THA for OA	Inability to obtain 6 week PROMIS scores
Lawrie et al. [28]	July 2020	III	172 (knees)	TKA	NR	NR	Primary TKA	Simultaneous bilateral TKA, lack of preoperative PROMIS scores, lack of 1 year follow-up PROMIS scores
Tang et al. [29]	Mar 2022	III	3667	TKA	66.4	43	Primary TKA with completion of PROMIS PF and KOOS-PS on same day	Did not complete any PROMIS or KOOS-PS, did not complete on same day
*Outcomes*								
Kagan et al. [30]	Mar 2022	II	91	TKA	63	54	Primary unilateral TKA, age > 40 years	TKA on contralateral knee within 6 months, any additional orthopedic, neurological, or visual conditions that could affect outcomes
Melnic et al. [31]	Oct 2022	III	1392	TKA	66	41	Primary unilateral TKA, completed PROMIS surveys pre- and postoperative	NR
Frye et al. [32]	Nov 2022	III	327 (96 MC, 70 CR, 161 PS)	TKA	MC: 64.3 CR: 63.4 PS: 66.3	MC: 46 CR: 42 PS: 43	OA, all pts undergoing TKA	NR
Christensen et al. [33]	Mar 2020	II	65	TKA	57.7	49	Primary TKA, end-stage OA, age < 65 years	NR

*pts* patients

### Validity

**Fig. 2:**
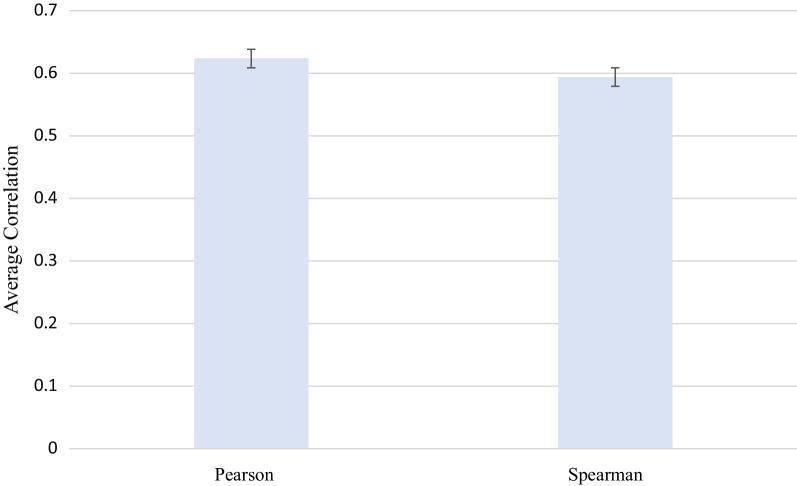
Weighted-average strength of the correlation between PROMIS and legacy for Pearson and Spearman correlation procedures. Data bars represent mean ± 1 standard error

Seven studies (*n* = 7011 patients) reported on PROMIS validity in TKA (Table 2) [21–23, 25, 27, 31, 34]. The weighted-average Pearson correlation coefficient was 0.62 [standard error (SE) = 0.06] and the weighted-average Spearman correlation comparing PROMIS domains with legacy PROMs in TKA patients was 0.59 (SE = 0.06) (Fig. 2). The direction of the correlation cannot be commented on as these were absolute value calculations. When evaluating individual PROMIS domains, the weighted-average correlation coefficient for PF was *r* = 0.79, *r* = 0.64 for PI, *r* = 0.30 for GMH, and *r* = 0.5 for GPH.

**Table 2:** Pearson and Spearman correlations between PROMIS and knee arthroplasty legacy instruments

Author	*N*	PROMIS Domain	Legacy	Correlation	*p*-Value
Khalil et al. [19]	875	PROMIS GPH	KOOS-JR	0.51^*^	< .001
Padilla et al. [20]	124	PROMIS PF	KOOS-JR	0.54^*^	< .01
		PROMIS PI	KOOS-JR	(−)0.64^*^	< .01
		PROMIS PIs	KOOS-JR	(−)0.63^*^	< .01
Heng et al. [23]	1003	PROMIS PF	KOOS-ADL	0.84^**^	NR
			KOOS-ADL Sports	(−)0.81^**^	NR
Austin et al. [21]	217	PROMIS GPH (preoperative)	M-SANE	0.28^**^	< .001
		PROMIS GPH (1–90 days)	M-SANE	0.4^**^	< .001
		PROMIS GPH (270–365 days)	M-SANE	0.65^**^	< .001
		PROMIS GMH (preoperative)	M-SANE	0.15^**^	< .001
		PROMIS GMH (1–90 days)	M-SANE	0.26^**^	< .001
		PROMIS GMH (270–365 days)	M-SANE	0.31^**^	< .001
		PROMIS GPH (preoperative)	KOOS-JR	0.62^**^	< .001
		PROMIS GPH (1–90 days)	KOOS-JR	0.6^**^	< .001
		PROMIS GPH (270–365 days)	KOOS-JR	0.7^**^	< .001
		PROMIS GMH (preoperative)	KOOS-JR	0.33^**^	< .001
		PROMIS GMH (1–90 days)	KOOS-JR	0.33^**^	< .001
		PROMIS GMH (270–365 days)	KOOS-JR	0.36^**^	< .001
Shim et al. [25]	721	PROMIS GPH	OKS	0.57^*^	< .001
		PROMIS GMH	OKS	0.14^*^	< .001
Nwanko et al. [29]	404	PROMIS GPH	BRS	0.32^*^	< .05
		PROMIS GMH	BRS	0.64^*^	< .05
Tang et al. [32]	3667	PROMIS PF	KOOS-PS	(−)0.79^*^	NR

^*^Denotes Pearson’s correlation coefficient

^**^Denotes Spearman’s correlation coefficient

### Floor and ceiling effects

**Fig. 3:**
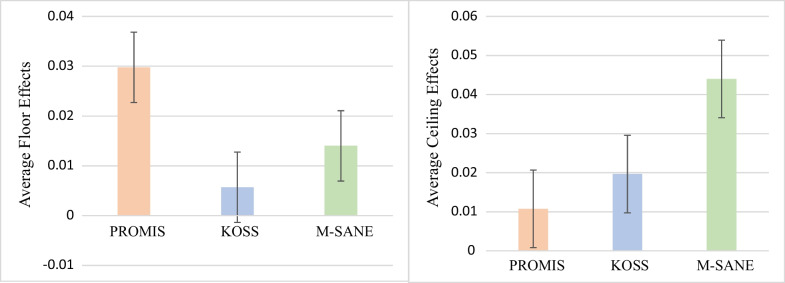
Results of floor (left) and ceiling (right) effects between PROMIS, KOOS-JR, and M-SANE. Data bars represent ± 1 standard error

Three studies reported on the floor and ceiling effects of PROMIS domains and legacy instruments (Table 3) [21, 23, 33]. PF had 0% floor and 0.13% ceiling effects, while PI had 8.5% floor and 0% ceiling effects. GPH had 0.1% floor and 0.2% ceiling effects, and GMH had 0.007% floor and 6.3% ceiling effects. Among PROMIS, KOOS-JR, and M-SANE there were no differences in weighted average floor effects [PROMIS: 0.03% (SE = 3.1) versus KOOS-JR: 0% (SE = 0.1) versus M-SANE: 0.01% (SE = 1.1); *p* = 0.25]. There were no differences in weighted ceiling effects between PROMIS and legacy instruments [PROMIS: 0.01% (SE = 0.7) versus KOOS-JR: 0.02% (SE = 1.4) versus M-SANE: 0.04% (SE = 3.5); *p* = 0.36] (Fig. 3).

**Table 3:** Floor and ceiling effects in PROMIS and legacy instruments

Author	*N*	Procedure	PROMIS domain	Timepoint	Floor (%)	Ceiling (%)
Khalil et al. [19]	875	TKA	PROMIS GPH	NR	0	0
Austin et al. [21]	217	TKA	PROMIS GPH	Preoperative	< 0.1	0
			PROMIS GPH	(1–90 days)	0	< 0.1
			PROMIS GPH	(270–365 days)	0	1.60
			PROMIS GMH	Preoperative	< 0.1	5.60
			PROMIS GMH	(1–90 days)	< 0.1	6.20
			PROMIS GMH	(270–265 days)	0	7.20
			KOOS-JR	Preoperative	1	0.20
			KOOS-JR	(1–90 days)	< 0.1	0.90
			KOOS-JR	(270–265 days)	1	4.80
			M-SANE	Preoperative	4	1.00
			M-SANE	(1–90 days)	0	0.90
			M-SANE	(270–265 days)	0	11.30
Lawrie et al. [31]	172	TKA, BPCI	PROMIS PF	Preoperative	0	0
			PROMIS PF	12 months	0	0
			PROMIS PI	Preoperative	0	0
			PROMIS PI	12 months	20	0
			Depression	Preoperative	13	0
			Depression	12 months	38	0
		TKA, non-BPCI	PROMIS PF	Preoperative	0	0
			PROMIS PF	12 months	0	0.50
			PROMIS PI	Preoperative	0	0
			PROMIS PI	12 months	14	0
			Depression	Preoperative	21	0
			Depression	12 months	38	0

### PROMIS responsiveness

**Fig. 4:**
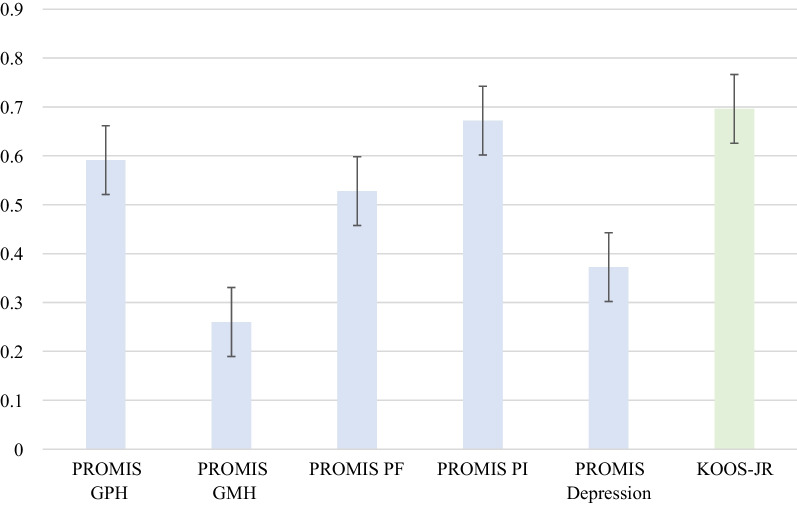
Weighted-average percentage of patients reaching MCID in PROMIS GPH, GMH, PF, PI, and depression scores. Data bars represent mean ± 1 standard error

Six studies on 3949 patients reported preoperative and postoperative PROMIS scores (Table 4) [21, 23, 28–30, 33]. For GPH, GMH, PF, and PI domains, weighted-average preoperative, < 3 months postoperative, and ≥ 6 months postoperative scores were calculated (Fig. 4). GPH, GMH, and PF all increased from baseline to < 3 months postoperative. (Table 4). These differences were statistically significant for GPH at < 3 months postoperative (baseline to < 3 months postoperative: 38.6 ± 5.3 to 42.4 ± 5.2, *p* = 0.002) and ≥ 6 months (baseline to ≥ 6 months postoperative: 38.6 ± 5.3 to 44.6 ± 5.7, *p* = 0.002). The change from baseline to ≥ 6 months postoperative was statistically significant for PF (baseline to ≥ 6 months postoperative: 36.7 ± 5.4 to 42.8 ± 7.4, *p* = 0.001). PI scores significantly decreased from baseline to postoperative (baseline to ≥ 6 months postoperative: 64.0 ± 6.0 to 53.6 ± 9.6, *p* = 0.001) Likewise, depression scores decreased from baseline to postoperative, but this was not found to be significant (baseline to ≥ 6 months postoperative: 47.9 ± 9 to 43.3 ± 8.8, *p* = 0.08). Changes from preoperative to postoperative GMH scores were not found to be statistically significant at < 3 months postoperative (baseline to < 3 months postoperative: 47.0 ± 6.6 to 47.3 ± 5.7, *p* = 0.2) and ≥ 6 months postoperative (baseline to: 47.0 ± 6.6 to 47.9 ± 5.5, *p* = 0.1).

**Table 4:** Preoperative and postoperative PROMIS scores in total knee arthroplasty studies

Author	Patient number	Procedure	Instrument	Preoperative	*F*/*u*	Postoperative	*p*-Value
Khalil et al. [19]	875	TKA	PROMIS GPH	38.5 ± 4.5	1 month	40.9 ± 4.6	< .001
			PROMIS GPH		3 months	42.2 ± 4.9	< .001
			PROMIS GPH		6 months	42.9 ± 5.1	< .001
			PROMIS GPH		12 months	43.2 ± 4.7	< .001
			PROMIS GMH	46.2 ± 5.2	1 month	46.2 ± 5.1	> .05
			PROMIS GMH		3 months	46.0 ± 4.9	> .05
			PROMIS GMH		6 months	46.4 ± 5.0	> .05
			PROMIS GMH		12 months	47.1 ± 4.2	> .05
Austin et al. [21]	217	TKA	PROMIS GPH	40.7 ± 6.9	1–90 days	42.8 ± 6.4	NR
			PROMIS GPH		270–365 days	47 ± 8.5	NR
			PROMIS GMH	50.0 ± 8.7	1–90 days	50.6 ± 8.7	NR
			PROMIS GMH		270–365 days	51.8 ± 8.9	NR
Frye et al. [28]	327	TKA	PROMIS GPH	MC: 38 ± 5.6 CR: 38.2 ± 6.5 PS: 36.8 ± 6.8	3 months	MC: 47.7 ± 7.4 CR: 46.6 ± 6.4 PS: 46.9 ± 7.3	0.69
			PROMIS GPH		12 months	MC: 52.1 ± 8.8 CR: 51.3 ± 7.5 PS: 51.1 ± 8.8	0.53
			PROMIS GMH	MC: 48.6 ± 9.0 CR: 47.8 ± 9.0 PS: 46.5 ± 8.8	3 months	MC: 51.8 ± 7.1 CR: 52.4 ± 8.7 PS: 51.4 ± 7.7	0.75
			PROMIS GMH		12 months	MC: 52.1 ± 7.4 CR: 51.3 ± 6.4 PS: 51.1 ± 9.7	0.76
Kagan et al. [27]	91	TKA	PROMIS PF CAT	38.7	6 weeks	39.2	0.41
			PROMIS PF CAT		3 months	44.1	< .001
			PROMIS PF CAT		6 months	46.4	< .001
			PROMIS PF CAT		12 months	47.3	< .001
			PROMIS PI CAT	61.7	6 weeks	58.1	0.001
			PROMIS PI CAT		3 months	54.2	< .001
			PROMIS PI CAT		6 months	52.2	< .001
			PROMIS PI CAT		12 months	50.7	< .001
Melnic et al. [26]	1392	TKA	PROMIS PF	36.8 ± 5.2	12 months	42.3 ± 7.2	< .001
			PROMIS GMH	47.6 ± 8.6	12 months	49.4 ± 9.1	< .001
Lawrie et al. [31]	172	TKA, BPCI	PROMIS PF	35.4 ± 5.8	12 months	42 ± 7.7	< 001
			PROMIS PI	64.2 ± 5.6	12 months	53.9 ± 9.8	< 001
			Depression	47.3 ± 8	12 months	43.3 ± 8.5	0.0026
		TKA, non-BPCI	PROMIS PF	35.9 ± 6	12 months	43.4 ± 8.4	< .001
			PROMIS PI	65.1 ± 6.4	12 months	55.5 ± 9.3	< .001
			Depression	48.5 ± 10	12 months	43.2 ± 9.1	0.0026

### MCID

Five studies reported on the MCID and the percentage of patients reaching MCID with PROMIS domains (Table 5) [21, 24, 26, 32, 33]. The weighted-average percentage of patients achieving MCID was 59.1% for GPH, 26.0% for GMH, 52.7% for PF, 67.2% for PI, and 37.2% for depression.

**Table 5:** PROMIS and legacy instrument MCID values and percentage of patient achievement

Author/year	Procedure	*N*	PROMIS domain	MCID	Percent achieving postoperatively ()	Timepoint
Khalil et al. [19]	TKA	875	PROMIS GPH	2.3	57	1 month
			PROMIS GPH	2.3	70	3 months
			PROMIS GPH	2.3	70	6 months
			PROMIS GPH	2.3	76	12 months
			KOOS-JR	6.6	65	1 month
			KOOS-JR	6.6	79	3 months
			KOOS-JR	6.6	84	6 months
			KOOS-JR	6.6	70	12 months
Shaw et al. [24]	rTKA	260	PROMIS GPH	3.4	33.20	1 month
			PROMIS GMH	4.5	23.60	1 month
			KOOS-JR	6.6	62.30	1 month
	Standard TKA	900	PROMIS GPH	3.4	36	1 month
			PROMIS GMH	3.8	26.70	1 month
			KOSS-JR	6.6	70.10	1 month
Darrith et al. [22]	TKA	872	PROMIS GPH	2.3	54.20	NR
			KOOS-JR	2.3	59.00	NR
Stiegel et al. [30]	TKA	50	PROMIS PF CAT	11.3	28	6 weeks
			PROMIS PI CAT	8.9	68	6 weeks
			Depression	4	14	6 weeks
Lawrie et al. [31]	TKA	172	PROMIS PF	5	60	12 months
			PROMIS PI	5	67	12 months
			Depression	5	44	12 months

Five studies on 3455 patients utilized PROMIS as an outcome metric (Table 6) [26, 28, 30, 31, 33]. These studies assessed outcomes such as the effectiveness of a certain implant, the effect of preoperative mental health on postoperative outcomes, and the feasibility of PROMIS in bundle-care payment improvement patients.

**Table 6:** PROMIS utilization as an outcome metric across total knee arthroplasty studies encompassed in this review

Author	Design	Pt population/intervention	*F*/*U*	Primary assessment	Main findings
Shaw et al. [24]	Prospective	TKA versus rTKA	1 month, 4–8 months	Comparison of evidence of improvement MCID in rTKA compared with standard total knee arthroplasty TKA	rTKA demonstrated comparable MCID achievement to standard TKA; no statistically significant difference in MCID achievement between rTKA and standard TKA
Frye et al. [28]	Prospective	TKA: MC versus CR versus PS	3 months, 12 months	Medial congruent polyethylene (MC) effect on patient outcomes after TKA	MC bearing resulted in similar or improved PROMIS, ROM, and patient satisfaction
Melnic et al. [26]	Retrospective	TKA	12 months	Comparison between patient-reported mental health and postoperative physical function following TKA	Patients with worse PROMIS GMH scores had worse PROMIS PF scores before and after surgery
Nwanko et al. [29]	Prospective	TKA	3 months	Preoperative resilience effect on 3-month postoperative outcomes	Resilience predicts postoperative knee function and general physical health in patients undergoing TKA
Lawrie et al. [31]	Retrospective	TKA	12 months	Feasibility and responsiveness of PROMIS in bundle payment care improvement patients undergoing TKA	PROMIS PF domain showed utility in assessing baseline status and changes in PROs over time in patients undergoing TKA, irrespective of insurance status

### Meta-analysis

A meta-analysis was performed on seven studies reporting correlations; statistical heterogeneity was found among studies (*I*^2^ = 98%, *p* < 0.0001). Overall, the meta-analysis showed significant difference between correlations (*p* < 0.0001).

### MINORS criteria

The mean MINORS score for all included studies was 19.3 ± 1.3 (range: 17 to 22) points (Table 1). All items assessed using the MINORS criteria demonstrated excellent inter-reviewer reliability with *k*-coefficient ≥ 0.7 in all items (Appendix 2 Table 9).

## Discussion

This is the first systematic review on the psychometric properties and use of PROMIS in assessing TKA outcomes. PROMIS has a moderate-to-strong correlation with the legacy instruments KOOS-JR, KOOS-ADL, KOOS-ADL Sports, OKS, M-SANE, and Brief Resilience Score (BRS), establishing strong criterion validity for PROMIS in TKA patients. PF consistently had strong to very-strong correlations across all compared legacy instruments. GMH was the most variable, with correlations ranging from very weak (*r* = 0.15) to strong (*r* = 0.70). This variability within the PROMIS umbrella indicates that certain PROMIS domains may not be as valid for the evaluation of TKA outcomes as others. Our results suggest that the global PROMIS domains (GMH more so that GPH) are less consistently correlated with legacy instruments, while the specific domains (PF and PI) are more consistently strongly correlated. This is likely due to the nonspecific nature of the global domains and their associated broad question banks.

Our study demonstrated 0% floor and ceiling effects for the majority of PROMIS domains (Table 3), showcasing the ability of PROMIS instruments to differentiate between patients both severely and minimally affected by knee arthritis and subsequent TKA. There were no differences found between PROMIS metrics and legacy instruments on pooled analysis of floor and ceiling effects, implying that PROMIS measures are equally effective as KOOS-JR and M-SANE at demonstrating measurement accuracy. PF and PI had insignificant floor and ceiling effects, falling below the 5–15% cutoff range. Similarly, both global PROMIS domains had low floor and ceiling effects. These findings are consistent with the general orthopedic literature: in a cross-sectional study of 94 patients with general knee pain, there were no floor or ceiling effects found with PF, and minimal floor and ceiling effects with KOOS-JR (3.4% and 1.1%, respectively) [36]. Conversely, in the only study that examined PROMIS depression, depression was found to have large floor effects (Lawrie: 21% preoperatively and 38% postoperatively) [33]. The elevated floor effects may indicate that depression may have limited responsiveness in patients who have a lower severity of depression and that its use is limited.

Responsiveness results were variable across different forms of PROMIS. Poor responsiveness was found in GMH and depression. Scores within these domains may be prone to fluctuation, as an individual’s baseline mental health score may be more likely to oscillate than one’s physical function due to mood changes unrelated to TKA. Regarding the global PROMIS domains, responsiveness was more robust at later follow-up (> 6 months postoperative). Responsiveness of an outcome measurement is crucial, as the ability of that tool to discern change through the course of treatment directly augments the ability of that tool to predict outcomes. The ability of PROMIS to capture the assessment of a TKA patient’s overall health while also accurately detecting changes in their knee function further supports its use in this population.

Notably, GPH, PF, and PI had high percentages of patients reaching MCID, with PI most effectively capturing clinical improvement, as evidenced by the achievement of MCID. GMH and depression were less able to showcase clinical improvement, with only 26% and 37.2% of patients achieving MCID, respectively. No statistical differences were found between PROMIS domain and the ability of the legacy instrument to capture clinical improvement. MCID values for PROMIS domains in TKA patients are listed in Table 7, and can be used by clinicians to predict and counsel a patient on how much they are expected to improve after TKA. MCIDs are valued in patient outcomes research as they serve as a standard for treatment success. Reporting MCIDs with PROMIS scores can lead to a more meaningful interpretation of outcome scores.

There are limitations to our study. First, there was some heterogeneity among the PROMIS domains used in this study. Of the seven studies that evaluated PF and PI, four used PF and PI CAT, one used the short form, and two were unspecified. Yet despite this mild heterogeneity, scores from the various forms were comparable, likely due to the common question bank from which they were generated. Another limitation relates to the small number of patients in the analyses of correlation coefficients for some of the PROMIS domains, which could affect the potential clinical significance of the correlation coefficients. The correlation coefficient for PI to KOOS-JR was found to be 0.64, based on a sample size of 124 patients. Despite the strong correlation identified in this study, the small sample size may limit overall generalizability. Additionally, two-thirds of our included 15 studies were retrospective in nature. None used any blinding methods, which may have resulted in selection bias.

## Conclusions

Our findings indicate that PROMIS forms are valid and reliable for use by knee arthroplasty surgeons and researchers to evaluate the clinical outcomes of patients undergoing TKA. Given the strengths of validity, responsiveness, ability to detect clinical improvement, and low floor and ceiling effects of both PROMIS PF and PI, we recommend those PROMIS domains be further utilized and studied in the TKA patient population, as they were found to be the most effective in this study. As a standardized method of PROM assessment, PROMIS can help create valid, reliable, and consistent interpretation of the TKA patient population across the orthopedic literature.

## Data Availability

The dataset(s) supporting the conclusions of this article is(are) included within the article (and its additional file(s)).

## Appendix

See Tables 7 and 8.

**Table 7:** Quality of included studies as quantifies by MINORS Quality Appraisal Tool

Authors, Year	Item 1		Item 2		Item 3		Item 4		Item 5		Item 6		Item 7		Item 8		Item 9		Item 10		Item 11		Item 12		Mean	% of max score
Khalil et al.	2	*2*	2	*2*	1	*1*	2	*2*	2	*2*	2	*2*	2	*2*	0	*0*	1	*1*	1	*1*	2	*2*	2	*2*	19	79
Padilla et al.	2	*2*	2	*2*	1	*1*	2	*2*	1	*1*	2	*2*	2	*2*	2	*2*	2	*2*	2	*2*	2	*2*	2	*2*	22	92
Heng et al.	2	*2*	2	*2*	1	*1*	2	*2*	2	*2*	2	*2*	2	*2*	2	*2*	0	*0*	0	*0*	2	*2*	2	*2*	19	79
Austin et al.	2	*2*	2	*2*	2	*2*	2	*2*	2	*2*	2	*2*	1	*1*	1	*0*	0	*0*	0	*0*	2	*2*	2	*2*	18	75
Shim et al.	2	*2*	2	*2*	2	*2*	2	*2*	1	*1*	2	*2*	2	*2*	2	*2*	1	*0*	0	*0*	2	*2*	2	*2*	19.5	81
Shaw et al.	2	*2*	2	*2*	2	*2*	2	*2*	2	*2*	2	*2*	1	*1*	0	*0*	2	*2*	2	*2*	2	*2*	2	*2*	21	88
Darrith et al.	2	*2*	2	*2*	1	*1*	2	*2*	2	*2*	2	*2*	2	*1*	1	*1*	0	*0*	0	*0*	2	*2*	2	*2*	17	71
Nwanko et al.	2	*2*	2	*2*	2	*2*	2	*2*	2	*2*	2	*2*	2	*2*	2	*2*	0	*0*	0	*0*	*2*	*2*	2	*2*	20	83
Stiegel et al.	2	*2*	2	*2*	2	*2*	2	*2*	2	*2*	2	*2*	2	*2*	1	*0*	1	*0*	0	*0*	2	*2*	2	*2*	19	79
Lawrie et al.	2	*2*	2	*2*	1	*1*	2	*2*	2	*2*	2	*2*	2	*2*	0	*0*	1	*1*	2	*2*	2	*2*	2	*2*	19.5	81
Tang et al.	2	*2*	2	*2*	1	*1*	2	*2*	2	*2*	2	*2*	2	*2*	0	*0*	0	*0*	1	*1*	2	*2*	2	*2*	18	75
Kagan et al.	2	*2*	2	*2*	2	*2*	2	*2*	2	*2*	2	*2*	2	*2*	2	*2*	0	*0*	0	*0*	2	*2*	2	*2*	20	83
Melnic et al.	2	*2*	2	*2*	1	*1*	2	*2*	2	*2*	2	*2*	2	*2*	0	*0*	0	*0*	1	*1*	2	*2*	2	*2*	18	75
Frye et al.	2	*2*	2	*2*	2	*2*	2	*2*	2	*2*	2	*2*	2	*2*	0	*0*	1	*1*	1	*1*	2	*2*	2	*2*	20	83

Roman values: reviewer 1; italic values: reviewer 2

*MINORS* Methodological Index for Non-Randomized Studies

*Scores out of 2 possible points with a maximum of 24 for comparative studies

**Table 8:** Inter-reviewer K-coefficient for agreement of MINORS criteria for quality assessment of included studies

Item number	Methodological item for nonrandomized studies	*K*-coefficient
1	A clearly stated aim	1
2	Inclusion of consecutive patients	1
3	Prospective collection of data	1
4	Endpoints appropriate to the aim of the study	1
5	Unbiased assessment of the study end point	1
6	Follow-up period appropriate to the aim of the study	1
7	Loss to follow-up less than 5%	0.76
8	Prospective calculation of the study size	0.75
9	An adequate control group	0.75
10	Contemporary groups	1
11	Baseline evidence of groups	1
12	Adequate statistical analysis	1

*MINORS* Methodological Index for Non-Randomized Studies, *K-Coefficient* Kappa coefficient for interrater agreement

## Abbreviations

PROMIS: Patient-Reported Outcome Measurement Information System
TKA: Total knee arthroplasty
PRISMA: Preferred Reporting Items for Systematic Reviews and Meta-Analyses
PROM: Patient-reported outcome measurement
MCID: Minimally clinically important difference
SE: Standard error
GPH: Global physical health
GMH: Global mental health
PF: Physical function
PI: Pain interference
WOMAC: Western Ontario and McMaster Universities Osteoarthritis Index
KOOS: Knee Injury and Osteoarthritis Outcomes Score
OKS: Oxford Knee Score
LEAS: Lower Extremity Activity Scale
CAT: Computer adaptive test
IRT: Item response theory
THA: Total hip arthroplasty
KOOS-JR: Knee Injury and Osteoarthritis Outcomes Score for Joint Replacement
M-SANE: Modified Single Assessment Numerical Evaluation
BRS: Brief Resilience Score
